# The pyrroloquinoline quinone biosynthesis pathway revisited: A structural approach

**DOI:** 10.1186/1471-2091-9-8

**Published:** 2008-03-27

**Authors:** Sandra Puehringer, Moritz Metlitzky, Robert Schwarzenbacher

**Affiliations:** 1 University of Salzburg, Department of Molecular Biology, Billrothstrasse 11, 5020 Salzburg, Austria

## Abstract

**Background:**

The biosynthesis pathway of Pyrroloquinoline quinone, a bacterial redox active cofactor for numerous alcohol and aldose dehydrogenases, is largely unknown, but it is proven that at least six genes in *Klebsiella pneumoniae *(PqqA-F) are required, all of which are located in the PQQ-operon.

**Results:**

New structural data of some PQQ biosynthesis proteins and their homologues provide new insights and functional assignments of the proteins in the pathway. Based on sequence analysis and homology models we propose the role and catalytic function for each enzyme involved in this intriguing biosynthesis pathway.

**Conclusion:**

PQQ is derived from the two amino acids glutamate and tyrosine encoded in the precursor peptide PqqA. Five reactions are necessary to form this quinone cofactor. The PqqA peptide is recognised by PqqE, which links the C9 and C9a, afterwards it is accepted by PqqF which cuts out the linked amino acids. The next reaction (Schiff base) is spontaneous, the following dioxygenation is catalysed by an unknown enzyme. The last cyclization and oxidation steps are catalysed by PqqC. Taken together the known facts of the different proteins we assign a putative function to all six proteins in PQQ biosynthesis pathway.

## Background

Pyrroloquinoline quinone (4,5-dihydro-4,5-dioxo-1H-pyrrolo-[2,3-f]quinoline-2,7,9-tricarboxylic acid: PQQ) is a water soluble, heat-stable, tricyclic ortho-quinone. It serves as redox cofactor for various bacterial dehydrogenases[1] providing unique redox-features. Among the best known examples of enzymes that utilize PQQ as a noncovalent cofactor are methanol dehydrogenase [2] and glucose dehydrogenase [3]. In general, ortho-quinone cofactors are involved in various biological reactions that range from oxidative deaminations to free-radical redox reactions [4]. PQQ was the first cofactor to be found in this cofactor-family, followed by the identification of tryptophan tryptophylquinone (TTQ), trihydroxyphenylalanyl quinone (topaquinone or TPQ), lysine tyrosylquinone (LTQ) and the copper-complexed cysteinyltyrosyl radical. This family is the third family of cofactors following pyridine nucleotide- and flavin-dependent cofactors [5]. Among the quinone family PQQ is unique in that it features a high mid point redox potential, in the range of 90 mV, as compared to TPQ with -150 mV, LTQ with -182 mV, TTQ with -150 mV and Flavin with -45 mV [6,7]. PQQ has provoked significant additional interest because of its presence in foods, its antioxidant properties and its role as a growth-promoting factor [8-10]. Although no enzymes in animals have been identified that exclusively utilize PQQ, oral supplementation of PQQ in nanomolar amounts increases the responsiveness of B- and T-cells to mitogens and improves neurologic function and reproductive outcome in rodents [4]. It has been shown to be essential for normal growth and development in animals but its suggested role as a vitamin in mammals has to be determined [11]. A recently published paper proposed AASDH as a PQQ-dependent enzyme in mammals [12]. However, this claim prooved to be incorrect and therefore the claim of PQQ as a vitamin should be considered premature [13,14].

Except for PQQ, quinone cofactors are linked to the polypeptide chain and derived post-translationally from Tyrosine (Tyr) and Tryptophan (Trp) residues encoded within their parental polypeptide chain [15]. For example in copper containing amine oxidases TPQ is derived from peptidyl tyrosine. PQQ is distinct from the other quinone synthetic groups in that it is biosynthesized independent of its site of action.

**Figure 1:**
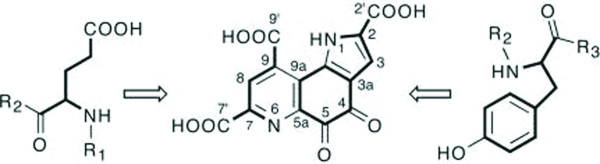
**Chemical structure of PQQ**. Chemical structure of PQQ (4,5-dihydro-4,5-dioxo-1*H*-pyrrolo-[2,3-*f*]quinoline-2,7,9-tricarboxylic acid) with atom nomenclature. All carbon and nitrogen atoms of PQQ are derived from conserved tyrosine and glutamate residues of the PqqA peptide. R_1 _and R_3 _represent the N- and C-terminal portions of PqqA, respectively. R_2 _represent a three-amino-acid linker between Glu and Tyr [26, 37, 38].

Several genes (PqqA-G) are required to derive PQQ from Glu and Tyr residues encoded in the precursor peptide PqqA (Figure 1).

*Klebsiella pneumoniae *needs six genes, seven genes are required in *Methylobacterium extorquens *(AM1) [16], whereas *Acinetobacter calcoaceticus *requires only four genes for PQQ synthesis. Interestingly, the homologous enzymes of PqqF and PqqG of *Methylobacterium extorquens *strain AM1 are missing. Although, much is known about the enzymes that use PQQ as a cofactor relatively little is known about its biosynthesis.

## Results and Discussion

### Enzymes and Reaction steps

**Figure 2:**
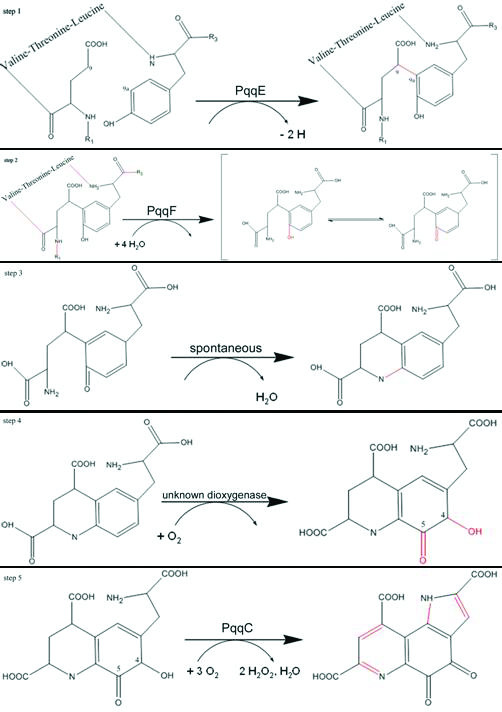
**Reaction Paythway**. The proposed mechanism of the PQQ biosynthesis pathway. Newly formed bonds are shown in red [17].

Due to the fact that Tyr and Glu are part of the peptide precursor PqqA the question arises how these two residues are cut out of the peptide and linked. C-C bond formation at atoms C9 and C9a (Figure 2, step **1**) is most probably one of the first reaction steps in order to link the two amino acids before the peptide bonds are cut. A likely candidate for catalyzing this reaction is PqqE, because it is the only enzyme in the pathway with significant sequence similarity (score -64, 16% sequence identity to Molybdenum cofactor biosynthesis protein A) to radical SAM proteins capable of catalysing C-C bond formation. After this reaction the now covalently linked amino acids Glu and Tyr are still linked to the PqqA-peptide backbone. Since PqqF is the only member with significant sequence similarity to proteases, it is most probably responsible for cleaving the four peptide bonds at R1 and R2 of Glu and R2 and R3 of Tyr (Figure 2, step **2**). After the linkage and proteolytic cleavage of the four peptide bonds the amino group N6 of Glu and the OH (C5a) of Tyr are primed to spontaneously form a Schiff-base reaction (Figure 2, step **3**). As a next possible reaction step two OH^- ^groups are added to atoms C4 and C5 in the Tyrosine ring which requires most likely a dioxygenase (Figure 2, step **4**). Yet, there is no protein with apparent sequence similarity to a dioxygenase in the PQQ operon. Candidates could be PqqB and PqqD, which however do not feature oxygenase similarities, or another oxygenase from the bacterium not exclusively used for PQQ-biosynthesis. The final step in the reaction has been elucidated and is catalyzed by PqqC [17]. The multi-step reaction includes a ring closure at N1 and the removal of eight electrons and eight protons from the intermediate to form PQQ (Figure 2, step **5**).

### PqqA

The small peptide PqqA is relatively conserved, although the length of the peptide varies between different organisms. The peptide is 23 amino acids in *K. pneumoniae *[18], 29 amino acids in *Methylobacterium extorquens *[16], 24 amino acids in *Methylobacillus flagellatum *[19] and 24 or 39 amino acids in *Pseudomonas fluorescens *[20]. The precursor residues glutamate and tyrosine are located in the conserved motif glu-X-X-X-tyr in the middle of the PqqA peptide. The secondary structure of this conserved motif is predicted to be a β-strand and both the glutamate and the tyrosine side chains would be oriented towards the same side, ideal for C-C bond formation at C9-C9a (Figure 2, step **1**). Different groups showed that mutations or frameshifts in PqqA lead to no or less production of PQQ [21]. In *A. calcoaceticus*, PQQ is not synthesized when glutamate is replaced by aspartate or when tyrosine is replaced by phenylalanine [21]. A shift in the reading frame of the small peptide of *K. pneumoniae *also inhibits regular PQQ synthesis [18]. These observations imply that the peptide serves as a complex precursor for PQQ synthesis. An alternative path for PQQ synthesis may exist in *M. extorquens*. In mutants lacking the gene for the small peptide, PQQ is synthesized, but at a reduced rate, i.e., 10–20% of that for the corresponding *M. extorquens *wild type [22]. These findings indicate that PqqA serves as the necessary precursor peptide. The fact that PqqA is in the same operon with all the other PQQ biosynthesis genes would indicate that every single enzyme of the pathway can only facilitate one reaction because only one substrate molecule is present. But on the protein level the expression of PqqA is 20-fold-higher than the expression of for example PqqC or PqqE [18].

### PqqB

PqqB is a 300 residue protein with a molecular mass of 33 kDa and a theoretical pI around 5.7.

Sequence analysis with FFAS [23], reveals a significant similarity to Ribonuclease Z (gi:16079441, Score of -54.2 and a sequence identity of 13%) and Metallo-beta-lactamases (gi: 102231667, Score of -22.5 and a sequence identity of 9%) were found by sequence analysis. PqqB contains a previously undescribed cysteine-rich sequence at the N-terminus, which is unique to PqqB sequences.

It has been reported that a knock out of PqqB produces small amounts of PQQ in the cytosol [24] and that no PQQ is secreted into the periplasm. The amount of PQQ in the cytosol was in an equimolar relationship to PqqC.

This finding indicates that PqqB is not directly required for PQQ biosynthesis but a carrier for PQQ and responsible for its transport across the plasma-membrane into the periplasm, where the bacterial dehydrogenases reside. Therefore, it is likely that PqqC needs an acceptor for PQQ because it can react with oxygen to generate free radicals [25]. PqqB could be required for the release of PQQ from PqqC and could act as PQQ-acceptor. Preliminary binding studies to determine whether PqqB actually binds to PqqC were undertaken with purified proteins. Our results indicate that PqqB neither binds to PqqC nor to the PqqC/PQQ-complex (data not shown).

**Figure 3:**
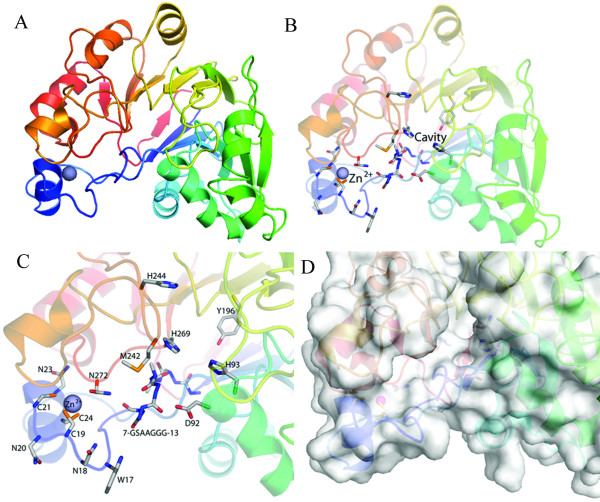
**Overview of the crystal structure of PqqB**. (A) General overview of the whole protein shown as cartoon. (B) Whole protein as cartoon with conserved residues shown in sticks. (C) Close up view of the putative active site near the Zinc atom. (D) Protein surface of the putative active site.

Recently, the crystal structure of PqqB (PDB: 1xto) was solved by Northeast Structural Genomics Consortium (NESG) and reveals a half-moon shaped molecule with the metallo-hydrolase/oxidoreductase fold (Figure 3).

PqqB contains a cavity lined by a number of strictly conserved residues. The cavity is adjacent to H93, H269 and Y169, and could easily receive a PQQ molecule, thereby supporting the carrier theory. PqqB also has a Zinc-finger at the N-terminal cysteine rich insertion (residues 5–24). The Zinc^2+ ^in this region is coordinated by three cysteines (C19, C21, and C24) and one asparagine (N272), all of which are strictly conserved. Near the N-terminus there is a conserved glycine rich stretch (7-GSAAGGG-13, Figure 3C) which is, like the whole N-terminal region, unique to PqqB sequences. Because glycine is a very flexible amino acid this short stretch could function as a hinge in a putative conformational change facilitating substrate binding, substrate recognition or ligand binding.

So far, the PqqB crystal structure reveals a putative PQQ binding site near the Zinc atom, however a detailed functional assignment could not be achieved and awaits further biochemical investigations using active site mutants.

### PqqC

**Figure 4:**
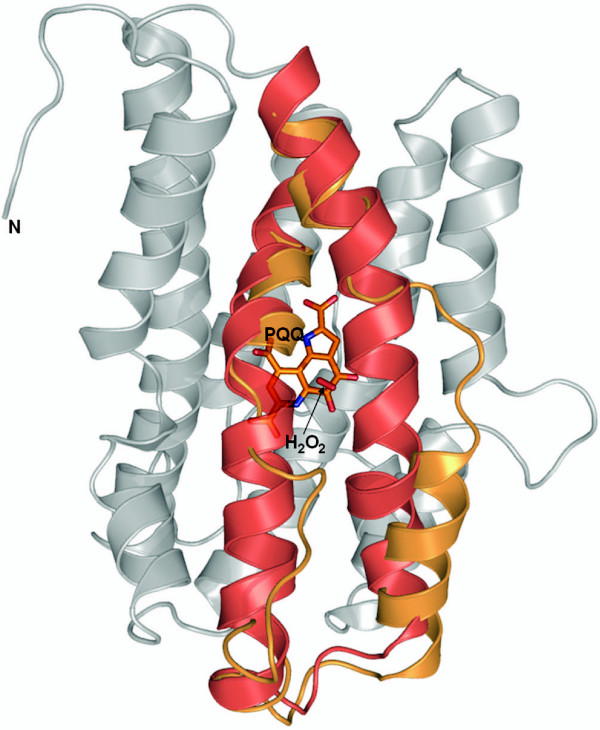
**Crystal structure of PqqC**. The two conformational states of PqqC (open: PDB ID 1otv; closed: PDB ID 1otw) shown in ribbon representation (grey). The moving parts are depicted as colored ribbons: open (orange) and closed (red) conformation. PQQ (orange) and putative hydrogenperoxide (red) are shown in sticks.

PqqC is a 250 residue protein with a molecular mass of 29 kDa and a theoretical pI of 6.9. It is a cofactorless oxidase which catalyzes the final step in the PQQ – pathway. The crystal structure of PqqC from Klebsiella pneumoniae [17] shows that the enzyme is a 58 kDa homodimer in which each monomer folds into a compact seven-helix bundle of six circular aligned helices, partly embracing a seventh hydrophobic helix. Analysis of the PqqC structure revealed that the seven α-helices provide the scaffold for a huge active site cavity. The cavity is lined with 42 mostly hydrophilic and aromatic residues that are highly conserved within PqqC proteins from different bacteria. The cavity shows a distinct overall positive charge, measures 9 Å × 13 Å × 23 Å and embraces a molecular surface volume of 2,200 Å^3 ^[17]. Two openings connect it to the outside. Upon substrate binding PqqC undergoes huge conformational changes. This rearrangement shifts the residues H154 and R157 into the active site. The largest shift occurs between residues 170 and 187, where the helix rotates about 90° around its long axis and shifts 2, 4, and 3.5 Å in three different directions [17]. Due to this shift these residues are in close proximity to PQQ, ready to coordinate the carboxylic group C7'. During this change Y175 moves 6.9 Å from a solvent exposed location to a position directly in the center of the molecule (Figure 4). The reaction catalyzed by PqqC has been identified [26] and it was found that it is an oxidase, which is unique in that it does not contain a redox active -metal or other -cofactor.

Former studies also described the purification and structure of the substrate as deduced by a number of spectroscopic and chemical methods. The substrate is 3a-(2-amino-2-carboxy-ethyl)-4,5-dioxo-4,5,6,7,8,9-hexahydro-quinoline-7,9-dicarboxylic acid – a fully reduced derivative of PQQ, which has not undergone ring cyclization [27]. Due to the fact that PQQ readily reacts with oxygen to generate free radicals [25], which are toxic to the cell, the question arises how PQQ is transported to its site of action, the periplasm, and how the release of PQQ from PqqC is facilitated. It is likely possible that another member of the pathway works as a carrier or that the enzyme that uses PQQ as a cofactor itself induces the PQQ-release. If no other enzyme is present, like in vitro, the reaction of PqqC is under the control of product inhibition and only measurable at single turnover conditions [17].

### PqqD

**Figure 5:**
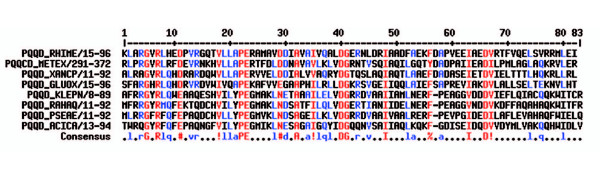
**Alignment of PqqD proteins from different organisms**. The alignment shows strictly conserved residues. Highly conserved residues are shown in red, low conserved residues are shown in blue, % indicates aromatic residues, ! indicates aliphatic residues and # indicates polar residues.

**Figure 6:**
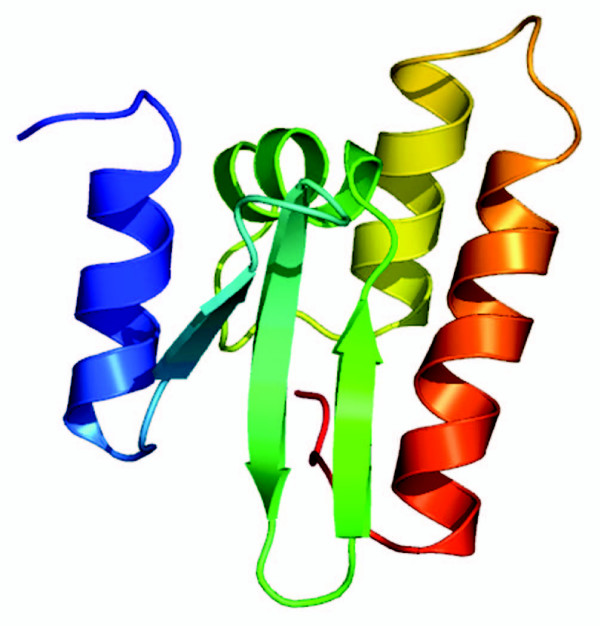
**3D model of PqqD**. Obtained from Robetta server (max. Z-score of 7.04)

PqqD is small protein with only 90 residues, a molecular mass of 10 kDa and a theoretical pI of 4.8. The alignment of PqqD proteins from different organisms shows strictly conserved residues (Figure 5) but few sequence homologies to other proteins can be found. Therefore, we can only speculate about its function, but it should catalyze one of the yet unassigned but required transformations. There are three possible functions for it: First, it could play a role in the release of PQQ from PqqC, second it could be involved in binding of PqqB to PqqC, third it could function as the dioxygenase in the pathway (Figure 2, step **4**). Since there is no significant sequence similarity to any of the known protein families we are not able to build a homology model. As a last resort, we did a de novo modelling using the robetta server [28,29], which indicates that the protein consists of 4 helices and 3 β-strands that form a β-sheet (Figure 6).

**Figure 7:**
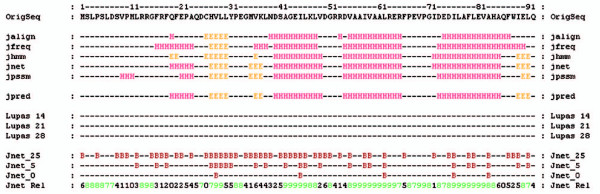
**Secondary structure prediction of PqqD**. Secondary structure prediction of PqqD from *Pseudomonas aeruginosa *made with Jpred at  [39] suggests that the protein is mostly helical with only short β-strands.

This result is consistent with the secondary structure prediction (Figure 7), but the real fold and structure await to be revealed.

### PqqE

**Figure 8:**
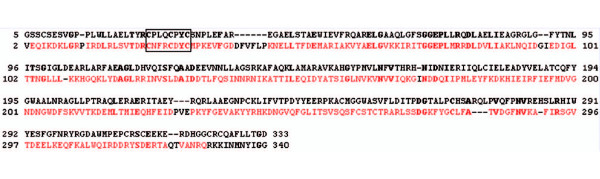
**PqqE Sequence alignment**. Alignment of PqqE (black) and Molybdenum cofactor6 biosynthesis protein A, PDB ID: 1tv7 (colored) from the FFAS-Server. The Alignment covers the whole sequence except for the last 50 residues. The FFAS score is -64 and the sequence identity is 16%, the boxed sequence is the iron-sulfur cluster coordinating motif.

PqqE is a 380 residue protein with a molecular mass of 43 kDA and a theoretical pI of about 5.7. An FFAS search revealed significant homologies with proteins from the radical SAM protein family (Figure 8).

The best homologue was Molybdenum cofactor biosynthesis protein A (PDB ID: 1tv7) which was, therefore, used as a template for homology modelling. Because the homologue has such a good score (6.7 times lower than the threshold of -9.5) we have a reliable model for amino acids 5 to 333 in PqqE.

**Figure 9:**
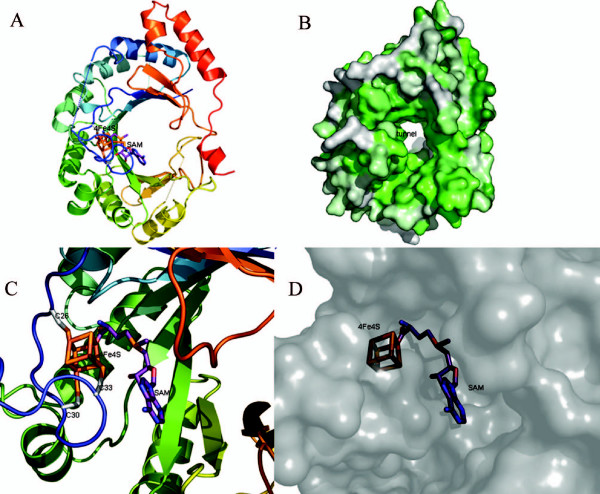
**Overview of the homology model structure of PqqE**. (A) General overview of PqqE shown as cartoon. (B) Surface view of the whole protein with the tunnel in the middle of the protein. Residues are color coded according to sequence conservation (white not conserved-green conserved). (C) Close up view of the putative active site near the iron-sulfur cluster, with conserved residues shown in sticks. (D) Protein surface of the putative active site, the cavity has a diameter of about 10 Å. The dashed lines are gap-regions in the alignments.

It is concluded that PqqE is a family member of radical S-adenosylmethionine (SAM) enzymes, because its sequence suggests that it contains a SAM domain with an iron-sulfur cluster. The four iron atoms of the cluster are coordinated by three cysteines (C26, C30, C33) from the conserved CxxxCxxC (C, Cys; x, any amino acid) motif of radical SAM enzymes and one atom is coordinated by the SAM (Figure 9C) PqqE-related enzymes often contain a Y at residue 27(Tyr) and have therefore a CxxxCxYC pattern.

The reaction catalyzed by PqqE is a radical driven C-C bond formation required to link the glutamate and tyrosine moieties at atoms C9 and C9a of PQQ. The exact mechanism is unknown but the analogy to radical SAM proteins implies the following mechanism:

The reduced 4Fe-4S cluster transfers an electron to the sulfur of SAM. The C5'-S^+ ^bond of SAM is cleaved, producing methionine and a highly oxidizing 5'-deoxyadenosyl radical. The radical abstracts a hydrogen atom from the tyrosine in PqqA, creating a tyrosine radical at position C9a. The radical reacts with atom C9 of glutamate leading to cyclization (Figure 2, step **1**).

The model protein has a globular shape, and features a tunnel through the whole protein and a cave at one end. This cave harbours the active site with the iron-sulfur-cluster and the bound SAM. The tunnel has a diameter of about 20 Å and the cave also about 20 Å (Figure 9).

For the reason that there is a tunnel to the active site and that PqqA forms a β-strand we propose that the PqqA moves through the tunnel to the iron-sulfur cluster where the Glutamate and Tyrosine side chains are then connected. At both ends of the tunnel and around the cave the residues are highly conserved and therefore most likely involved in substrate recognition and orientation.

### PqqF

**Figure 10:**
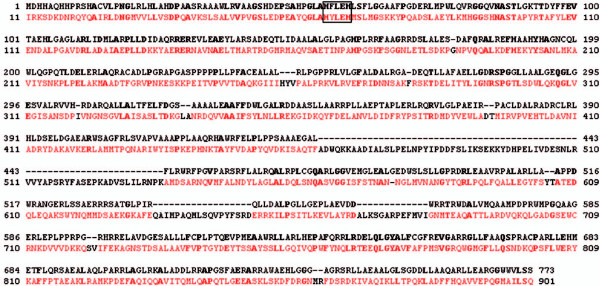
**Alignment of PqqF and pitrilysin**. Amino acid-sequence Alignment of PqqF (black) and pitrilysin from *Escherichia coli*, PDB ID: 1Q2L (colored), the zinc binding motif (HxxEH) is boxed. The FFAS score is -136 and the sequence identity is 20%.

**Figure 11:**
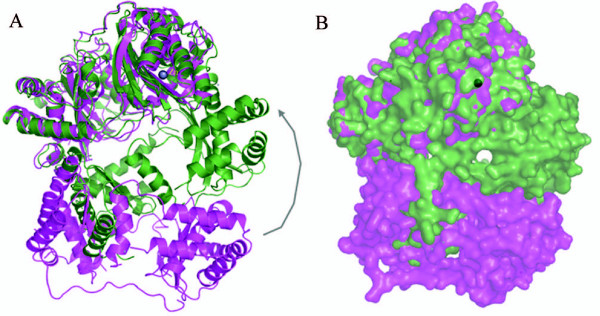
**Homology model structure of the open form of PqqF superposed with the closed form**. (A) Overview of the whole molecule (cartoon). (B) Overview of the whole molecule (surface)

PqqF is the biggest of the proteins in the pathway. It is about 760 residues long and has a molecular weight of 84 kD and a theoretical pI of 8.7. According to FFAS pitrilysin, a metalloendopeptidase from *Escherichia coli *(PDB ID: 1Q2L), shows the best similarity and was, therefore, used as a template to create a homology model for the open form of PqqF (Figures 10 and 11).

Like for PqqE the quality of the model is very high due to the good score (14.3 times lower than the threshold of -9.5) and sequence identity. There are three gaps of different size in the alignment (residues Ala464-Lys535, Gln633-Asp650, Ala670-Ile682) where our protein has no homology to the pitrilysin sequence.

PqqF and human insulin-degrading enzyme (IDE) (a metalloendopeptidase) share 17% sequence identity and a score of -128. The closed form with bound amyloid-β (PDB: 2G47) was used to create a model for the enzyme with bound substrate. Here the quality of the model is also very high due to the good score and sequence identity. (Alignment not shown).

Hence, PqqF is most likely a metalloendopeptidase involved in processing of the tyrosine and glutamate of PqqA at R1–R3 (Figure 1, Figure 2) with a Zinc centre in its active site. Its sequence has homologies (sequence identities of 15–20%) to insulin-degrading enzymes and protease III of Bacteria.

The Zinc ion is located near a reversed active site motif of thermolysin (HxxEH instead of HExxH) and an additional glutamate (E136). Because of earlier experiments from Becker and Roth [30] the two histidines are crucial for binding of the Zn^2+^and the glutamates are required for catalytic activity. Through its similarity to the insulin-degrading enzymes an analogous mechanism of binding and processing of the substrate (PqqA) is proposed. The fact that IDE is able to recognize different substrates suggests that PqqF could recognize and cleave all four peptide bonds in PqqA.

It is proposed that the protein has two conformational states, an open form and a closed form. In the open state the protein has two bowl-shaped domains with conserved residues on both termini. These termini attach when the substrate binds and the protein forms a cavity in the inside. This conformational change requires a shift of the outer helices of about 40 Å (Figure 11). This is called the closed state. When the substrate binds, the N-terminus (with conserved negatively charged amino acids) and C-terminus (with conserved positively charged amino acids) close in to form a triangular prism shaped chamber (base dimensions of 35 × 34 × 30 and a height of 36 Å with an approximate volume of 1.3 × 10^4 ^Å^3^) and trap the PqqA-peptide inside this cavity [31]. Now the peptide is cut at four positions; in this process several reorientation steps are requires in order to present the individual peptide bonds for cleavage in the active site. It is still unclear how the protein facilitates the cleavage because all four bonds are cleaved by the same active site. Maybe the mechanism is similar to the mechanism of the insulin degrading enzyme where the enzyme uses size and charge distribution of the cavity selectively to entrap structurally diverse polypeptides [31].

## Conclusion

The first step of the whole pathway (Figure 2) is the expression of PqqA as a precursor for PQQ. PqqA is recognised by PqqE, which links the C atoms from the glutamate and tyrosine to become C9 and C9a of the final product PQQ (Figure 1, Figure 2) and, therefore, enables recognition and acceptance of the modified PqqA by PqqF. In step two PqqF afterwards cuts out the linked amino acids. The following Schiff base reaction is spontaneous, the following dioxygenation is catalysed by an unknown enzyme. To finally finish the product PqqC facilitates the last cyclization and oxidation steps.

The knowledge of the proteins in the PQQ biosynthesis pathway today can be split into four categories: entirely unknown (PqqD), functional information based on mutational studies (PqqA), structural and functional information based on sequence homology (PqqE and PqqF), structural and functional information based on crystal structure (PqqB), structural and functional information based on crystal structure and experimental evidence (PqqC). PqqA is known to be the precursor of PQQ. For PqqB the crystal structure is known but the function still remains unclear and awaits further experimental characterisation. We are planning to construct mutants in order to study the importance of the Zinc-finger and conserved residues in the active site cavity for PqqB function. The structure and function of PqqC are well characterised, but the exact reaction mechanism for oxygen activation still needs to be revealed. About PqqD nearly nothing is known, so we can only speculate about its function. The fact that the genes for PqqC and PqqD are fused in some organisms (for example in *M. extorquens*) suggests a possible function in the release of PQQ from PqqC. Alternatively, PqqD could be the missing oxygenase in the pathway (Figure 2, step **4**). For PqqE we were able to construct a reliable homology model and derive hints about its function from the homologous radical SAM proteins. Hence, PqqE most likely forms the C-C bond between C9 and C9a using a radical SAM cofactor. Through the similarity of PqqF to metalloendopeptidases like Pitrilysin and IDE we can propose the reaction catalysed by PqqF as four cleavages in the peptide-backbone of PqqA to cut out the Glutamate and the Tyrosine-residues. For these two proteins the functional details await to be revealed by x-ray crystallography as well as biophysical studies and mutagenesis analyses.

In the future the following questions will have to be addressed: How does PqqE link the Glutamate and the Tyrosine in the peptide precursor PqqA? How does PqqF recognize four different cleavage sites with only one catalytic center? Which enzyme facilitates the oxygenation step (Figure 2, step **4**)? Is it performed by an unknown enzyme possibly PqqB, PqqD or an enzyme not coded in the PQQ-operon? Does the crystal structure of PqqD reveal a new fold and how many new reaction mechanisms are still waiting to be discovered?

## Methods

### Homology modeling

The Protein sequence was submitted to profile – profile sequence searches with the FFAS server [32].

The Proteins with the best sequence identity of the FFAS-search was used as a template for modelling the structures with the SCWRL-Server [32-34] (default settings with conformation of conserved residues were retained and no optimization was done with these). The conserved residues were identified with ConSurf-Server [35,36] and displayed using PyMOL software (DeLano Scientific). Through superposing we identified the putative active-site residues. Models were superposed using CCP4 suite [16]. Graphics and figures have been prepared using PyMOL.

## Authors' contributions

SP carried out the crystallographic studies on PqqC, MM made the homology models and drafted the manuscript. SP and MM worked out the reaction pathway and the functions of the involved enzymes. RS conceived of the study, and participated in its design and coordination. All authors read and approved the final manuscript.
